# Photothermal therapy via a gold nanoparticle-coated stent for treating stent-induced granulation tissue formation in the rat esophagus

**DOI:** 10.1038/s41598-021-90182-x

**Published:** 2021-05-18

**Authors:** Young Chul Cho, Jeon Min Kang, Wooram Park, Dong-Hyun Kim, Ji Hoon Shin, Do Hoon Kim, Jung-Hoon Park

**Affiliations:** 1 Departments of Radiology and Research Institute of Radiology, Asan Medical Center, University of Ulsan College of Medicine, 88 Olympic-ro 43-gil, Songpa-gu, Seoul, 05505 Republic of Korea; 2 Biomedical Engineering Research Center, Asan Institute for Life Sciences, Asan Medical Center, 88 Olympic-ro 43-gil, Songpa-gu, Seoul, 05505 Republic of Korea; 3 Department of Biomedical-Chemical Engineering, The Catholic University of Korea, 43 Jibong-ro, Bucheon-si, Gyeonggi 14662 Republic of Korea; 4 Department of Radiology, Feinberg School of Medicine, and Robert H. Lurie Comprehensive Cancer Center, Northwestern University, Chicago, IL 60611 USA; 5 Departments of Gastroenterology, Asan Medical Center, University of Ulsan College of Medicine, 88 Olympic-ro 43-gil, Songpa-gu, Seoul, 05505 Republic of Korea

**Keywords:** Gastrointestinal diseases, Nanomedicine, Biomedical engineering

## Abstract

Minimally invasive therapies using stent technology are currently limited by stent-induced granulation tissue formation adjacent to the stent. The effectiveness of photothermal therapy (PTT) using a gold nanoparticle (AuNP)-coated stent for treating stent-induced granulation tissue formation in the rat esophagus was investigated. All experiments were approved by the animal research committee of our institution. An AuNP-coated, self-expandable metallic stent (SEMS) was produced to conduct PTT under near-infrared laser irradiation. Forty rats were randomly divided into four groups (10 rats each). The animals in group A (non-coated SEMS) and group B (AuNP-coated SEMS with local heating at 65 °C at 4 weeks) were sacrificed 4 weeks after stent placement. The rats in group C (AuNP-coated SEMS with local heating at 65 °C at 4 weeks) and group D (AuNP-coated SEMS with local heating at 65 °C at 4 and 8 weeks) were sacrificed 8 weeks after stent placement. The effectiveness of local heating was assessed by histopathology. All procedures were successful in all of the animals. Seven rats were excluded because of stent migration (n = 2) and death (n = 5). Granulation tissue formation-related variables were significantly higher in group A than in groups B–D (all *p* < 0.05). Heat-shock protein 70 (HSP70) and TUNEL expression were significantly lower in group A than in groups B–D (all *p* < 0.05). Granulation tissue formation-related variables were significantly higher in group C than in groups B and D (all *p* < 0.05). PTT using AuNP-coated SEMS successfully treated granulation tissue formation after stent placement in the rat esophagus.

## Introduction

Esophageal stenting using a self-expandable metallic stent (SEMS) is currently the most common therapeutic strategy for treating malignant and benign diseases^1–3^. The use of SEMS devices is significantly limited however by malignant and/or benign tissue growth through the stent meshes after placement, which can lead to newly developed stricture formation and recurrent symptoms^2–5^. Drug-eluting, biodegradable, and nano-functionalized stents have been investigated as possible avenues to overcome this complication but have not been successful in treating stent-induced malignant and/or benign tissue formation^6–12^.

Photothermal therapy (PTT) offers a potential therapeutic strategy for cancer cells without significant collateral damage^13,14^. Gold nanoparticles (AuNPs) have excellent absorption properties in the near-infrared (NIR) spectrum of biological window and their PT-converting efficiency has been studied previously as a possible biomedical application for targeting different diseases^13–18^. Recently, AuNP-based functionalized stents were introduced to locally treat cancer cells or tissue hyperplasia adjacent to stented non-vascular luminal organs^11,12,19,20^. Park et al. have reported in rat models that locally applied temperature increases (50 to 65 °C) successfully suppressed tissue hyperplasia after esophageal and gastroduodenal stent placement^11,20^. We hypothesized that AuNP-coated SEMS could be used for localized PTT under NIR laser irradiation. We speculated that this approach would treat stent-induced granulation tissue formation after SEMS placement through the induction of PT-induced cell apoptosis and activation of heat-shock proteins (HSPs). We here investigated the feasibility of this therapy in the rat esophagus.

## Materials and methods

### Study design

**Figure 1:**
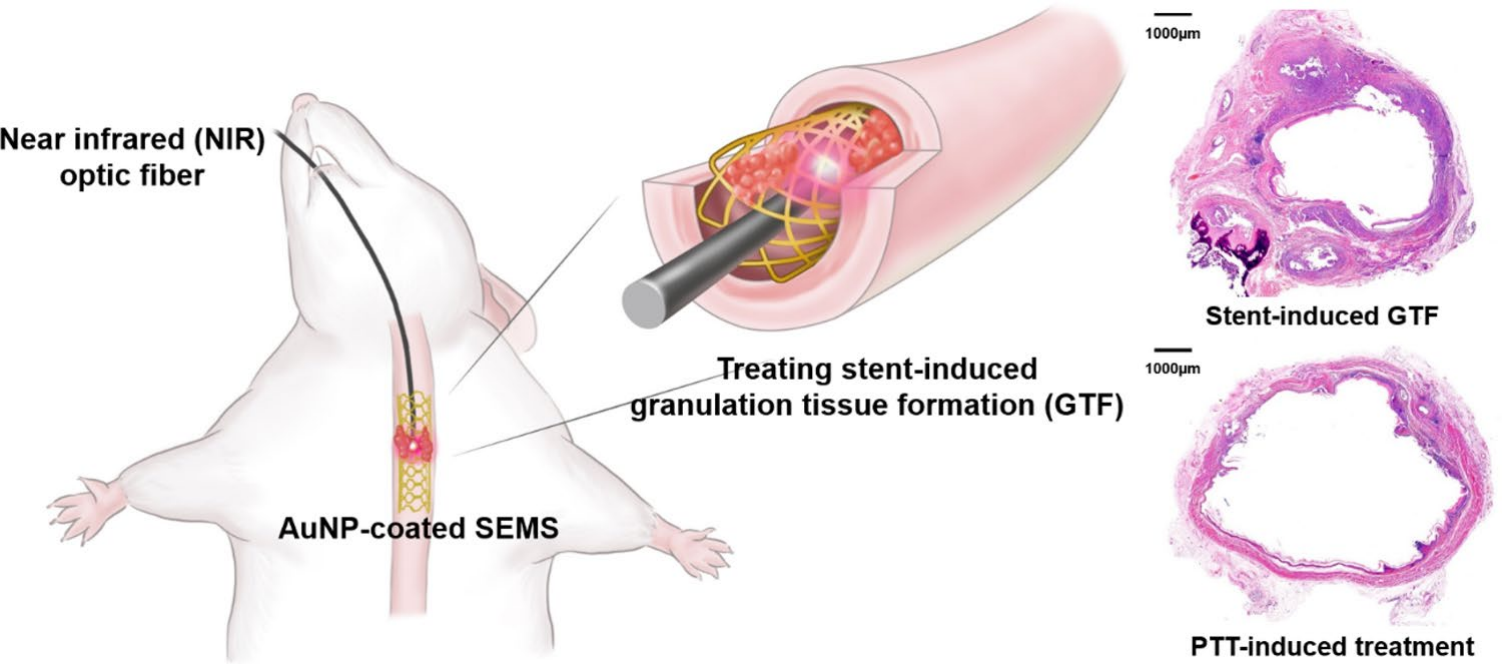
Schematic illustration of the photothermal therapy (PTT) method using a gold nanoparticle (AuNP)-coated self-expandable metallic stent (SEMS) and representative hematoxylin and eosin images shows PTT therapeutic effects (magnification, × 1.25).

The Institutional Animal Care and Use Committee of the Asan Institute for Life Sciences (Seoul, Korea) approved all of the experiment protocols and animals used in this study (2019-13-056). All experiments were performed in accordance with relevant guidelines and regulations. The study was carried out in compliance with the ARRIVE guidelines. The number of animals was prospectively calculated according to the previous report^6^. In total, 40 Sprague–Dawley male rats (300–350 g; Orient Bio, Seongnam, Korea) underwent SEMS placement into the esophagus. AuNP-coated SEMSs were prepared as described previously^11,20^. The animals were divided into four groups. Group A (n = 10) received non-coated SEMS. Group B (n = 10) received AuNP-coated SEMS with local heating at 65 °C at 4 weeks. All rats in groups A and B were sacrificed 4 weeks after stent placement. Two additional groups were included to evaluate any rebound effects of local heating. Group C (n = 10) received AuNP-coated SEMS with local heating at 65 °C at 4 weeks and then housed until 8 weeks. Group D (n = 10) received AuNP-coated SEMS with local heating at 65 °C at both 4 and 8 weeks. All rats in groups C and D were sacrificed 8 weeks after SEMS placement for histopathological analysis (Fig. 1). All animals were sacrificed by administering inhalable pure dioxide. The body weight of each rat was measured weekly until they were sacrificed. All animals were housed at one per cage in a room with a 12-h contrast cycle at 24 ± 1 °C with a relative humidity of 55 ± 10%. Standard rodent chow and water were provided ad libitum. All animals were acclimated for at least 1 week prior to conducting the experiments.

### Cytotoxicity of stent samples

The cytotoxicity of the control, polydopamine (PDA) coated, and AuNP coated wires was analyzed using a standard Cell Counting Kit-8 (CCK-8). L929 and 293 cells were seeded into 96-well plates at a concentration of 1 X 104 cells/well, respectively. After incubation at 37 °C for 24 h, each wire was placed on the different well for 1, 12, 24, 36, and 48 h, respectively, and 10 μl of CCK-8 solution was added to the 96-well plate and incubated. Cell viability was evaluated by measuring the absorbance of each well at 450 nm using a microplate reader. All experiments were repeated three times and results were averaged.

### Stent placement in the rat esophagus

For stent placements in the esophagus, the rats were anesthetized via an intramuscular injection of 50 mg/kg zolazepam and 50 mg/kg tiletamine (Zoletil 50; Virbac, Carros, France) and 10 mg/kg xylazine (Rompun; Bayer HealthCare, Leverkusen, Germany). A 0.014-inch guidewire (Transcend; Boston Scientific, Watertown, MA) was inserted through the mouth and negotiated into the stomach under fluoroscopic guidance. A customized 6-French (Fr) sheath and dilator were then advanced into the lower esophagus through the guide wire. With the sheath left in place, the dilator and the guidewire were removed. A stent in a compressed state was loaded into the sheath and placed in the esophagus using a pusher catheter. The stent was deployed at the level of the mid-thoracic esophagus under continuous fluoroscopic monitoring.

### Photothermal therapy under near-infrared irradiation

PTT under NIR irradiation was performed at both 4 weeks (groups B, C, and D) and 8 weeks (group D) after stent placement in the experimental rats. For NIR laser irradiation during the in vivo fluoroscope-guided procedure, a 1-mm diameter fiber-coupled NIR (808 nm) diode-laser (OCLA LASER, NDLUX Inc., Anyang, Korea) was inserted into a 6-Fr sheath with a radiopaque tip for visualization by fluoroscopy. Using fluoroscopic guidance, the sheath and optic fiber were advanced through the mouth into the middle portion of the stented esophagus. NIR laser irradiation was applied for 60 s in all rats. After stent placement and local heating, all animals were intramuscularly injected with 0.05 mg/kg of buprenorphine (Renophan; Hanlim Pharmaceutical, Seoul, Korea) every 6 h for pain control for 48 h. All animals were monitored until recovery from anesthesia and were then returned to their cages.

### Histological analysis

Surgical exploration of the entire esophagus and stomach was followed by gross examination to determine possible esophageal injury after stent placement or irradiation. Tissue samples were fixed in 10% neutral buffered formalin for 24 h, then embedded in paraffin and sectioned. The stented esophagus was sectioned transversely in the middle area. The slides were stained with hematoxylin and eosin (H&E) and Masson’s trichrome (MT). Histological evaluation using H&E was based on the degree of submucosal inflammatory cell infiltration, the number of epithelial layers, the thickness of the submucosal fibrosis, and the tissue-hyperplasia-related percentage of the esophageal cross-sectional area of stenosis calculated as 100 × (1 − [stenotic stented area/original stented area])^6,11^. The degree of inflammatory cell infiltration was subjectively determined according to the distribution and density of the inflammatory cells, i.e. graded as 1, mild when there was occasional visible infiltration of single leukocytes; 2, mild-to-moderate when there was patchy infiltration of leukocytes; 3, moderate when coalescing leukocytes made individual loci indistinguishable; 4, moderate-to-severe when there was diffuse infiltration of leukocytes throughout the submucosal layer; and 5, severe when there was diffuse infiltration with multiple necrotic foci^21^. The collagen-deposition level was subjectively determined with the following scores: 1 = mild, 2 = mild to moderate, 3 = moderate, 4 = moderate to severe, and 5 = severe. The esophagus was analyzed histologically using a BX51 microscope (Olympus, Tokyo, Japan). Image-Pro Plus 6.0 software (Media Cybernetics, Silver Spring, MD) (available at https://www.mediacy.com/imageproplus) was used for the measurements. The histological findings were based on the consensus of three observers who were blind to the experimental groups.

### Immunohistochemistry

Immunohistochemistry (IHC) was performed on paraffin-embedded sections using terminal deoxynucleotidyl transferase mediated dUTP nick and labeling (TUNEL) (Apoptotag kit, Biogene, Darmstadt, Germany) and HSP70 (1:1000; Abcam, Cambridge, UK) primary antibodies. The sections were visualized using a BenchMark XT IHC automated immunohistochemical stainer (Ventana Medical Systems, Tucson, AZ). The degree of TUNEL and HSP70 positive deposition was determined subjectively (1 = mild, 2 = mild to moderate, 3 = moderate, 4 = moderate to severe, and 5 = severe). IHC findings were also based on the consensus of three observers who were blind to the experimental groups.

### Statistical analysis

Data were expressed as a mean ± standard deviation (SD). Differences between the groups were analyzed using Kruskal–Wallis or Mann–Whitney U test, as appropriate. A p-value of < 0.05 was considered statistically significant. Statistical analyses were performed using SPSS software (version 24.0; SPSS, IBM, Chicago, IL) (available at https://www.ibm.com/analytics/spss-statistics-software).

## Results

### Stent placement and PT-mediated local heating

**Figure 2:**
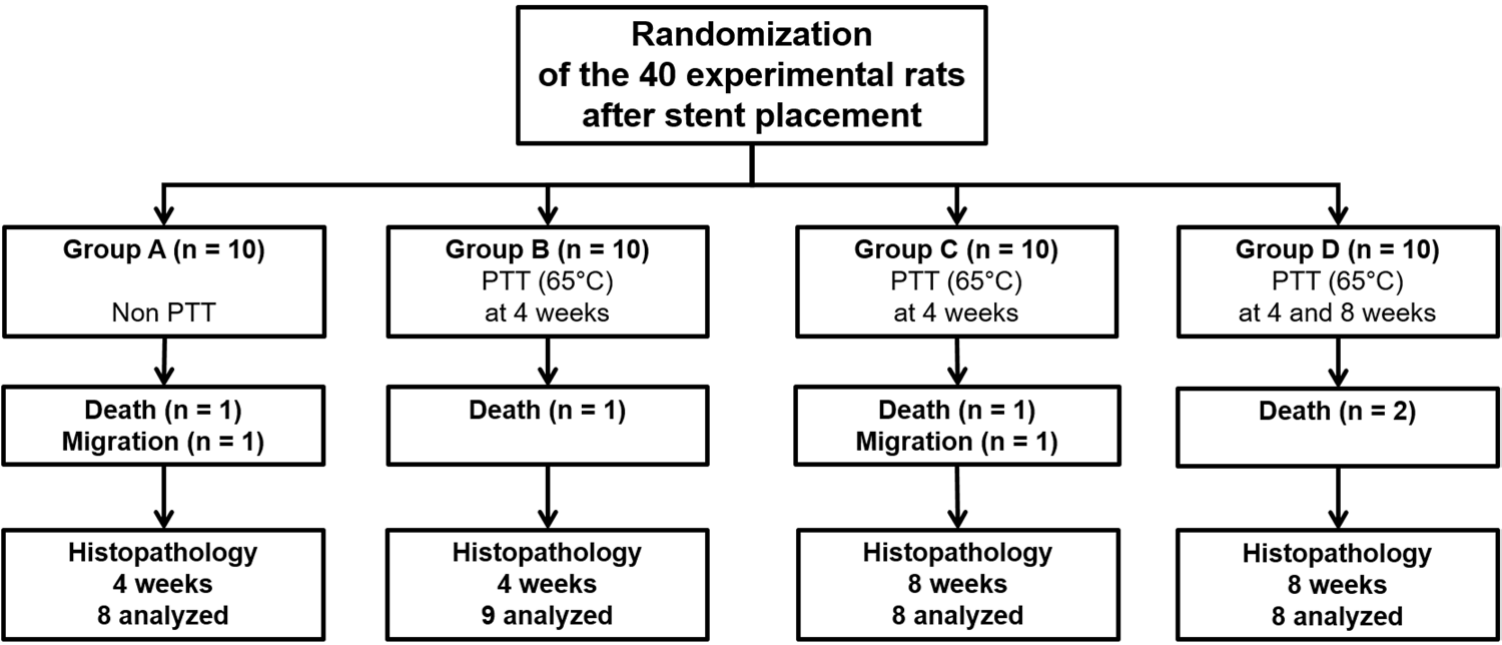
Flow diagram showing the randomization process and study follow-ups. Note. PTT; photothermal therapy.

Stent placement and PTT were technically successful in all the experimental rats. Five of 40 (12.5%) rats (one each in groups A-C and two in group D) died after stent placement due to a hemorrhage caused by the stent barbs at 1–3 days after placement. The AuNP-coated SEMS migrated into the stomach in two rats (one each in groups A and C) within 10 days of placement. The remaining 33 (82.5%) rats survived until the end of the study without stent-related complications (Fig. 2).

Although the body weights of the rats decreased at 1 week after stent placement and the PTT procedure, this did not significantly affect any of these animals in terms of their condition and behavior. There were also no significant differences between the groups in terms of the body weights after stent placement and the PTT procedure (*p* > 0.05) (Supplementary Fig. 1).

The cytotoxicity results showed that cell death was not observed, which indicated that the control, PDA-coated, and AuNP-coated stents were nontoxic, as shown in Supplementary Fig. 2.

### Histological findings

**Figure 3:**
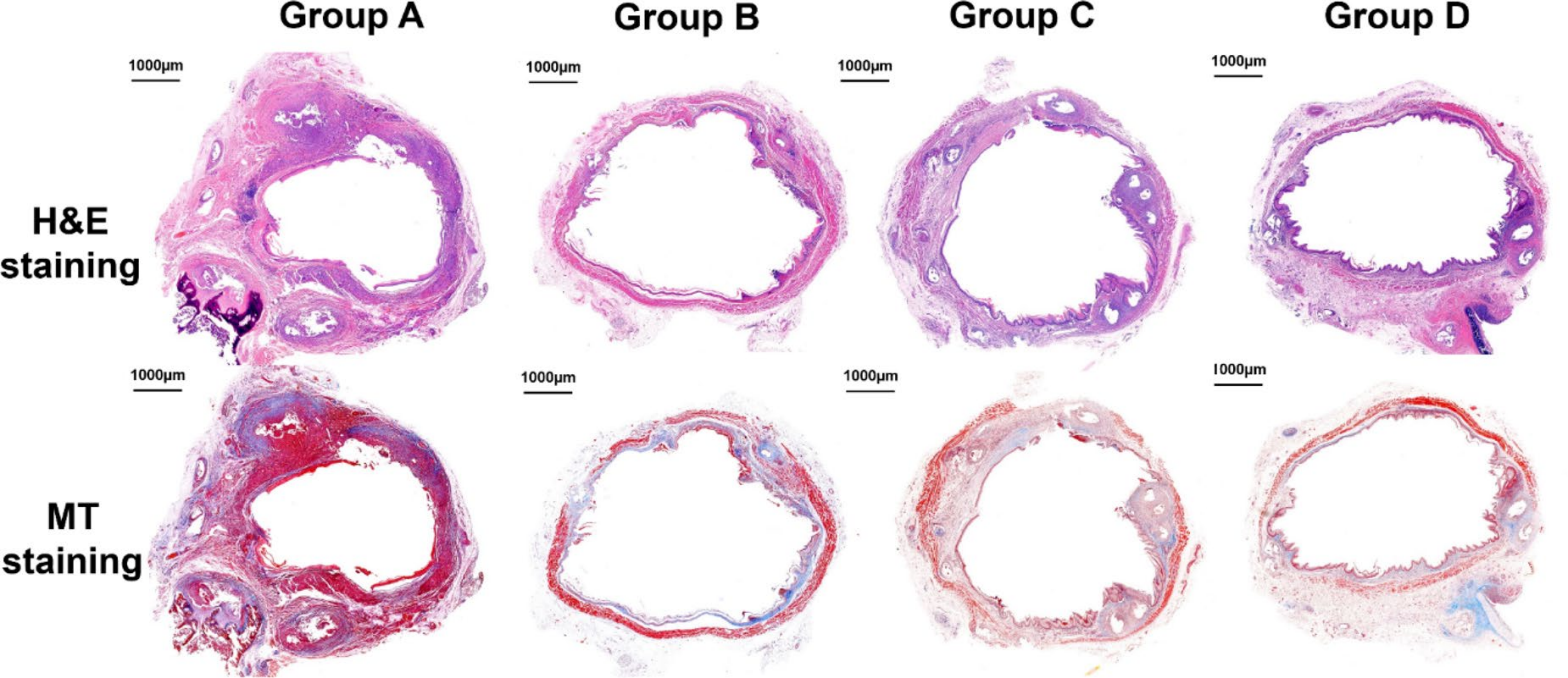
Representative microscopic images of histological sections obtained at 4 weeks (groups **A** and **B**) and 8 weeks (groups **C** and **D**) after stent placement. Hematoxylin and eosin and Masson’s trichrome stained sections are shown (magnification, × 1.25).

**Figure 4:**
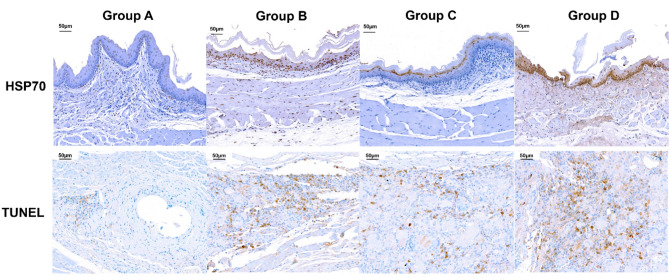
Representative microscopic images of immunohistochemistry sections obtained at 4 weeks (groups **A** and **B**) and 8 weeks (groups **C** and **D**) after stent placement. HSP70 and TUNEL expression were significantly increased in the heated groups compared to the control group (magnification, × 20).

**Figure 5:**
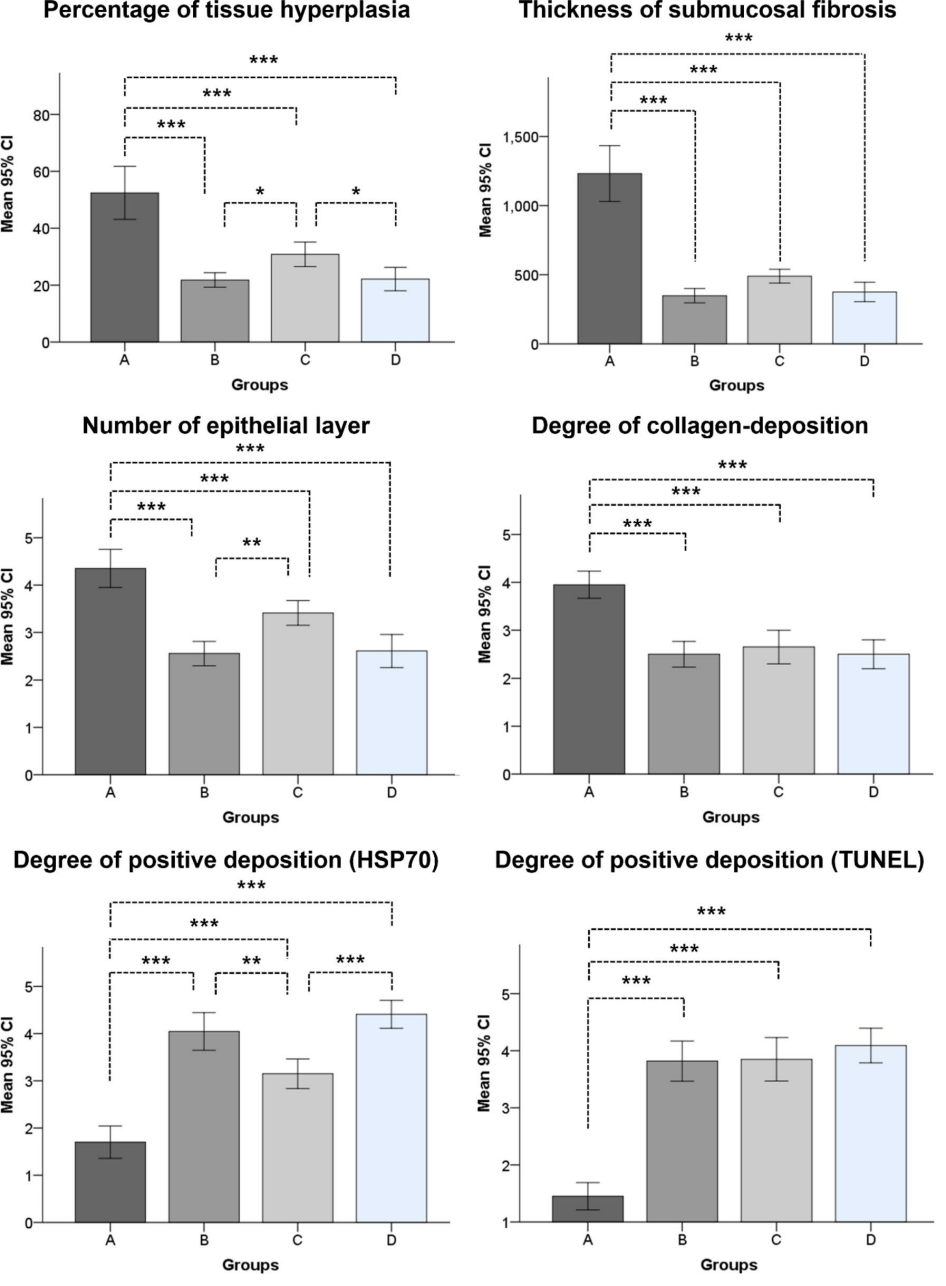
Histopathological findings for the stented rat esophagus at 4 and 8 weeks after stent placement in groups (**A**-**D**); *p < 0.05, **p < 0.01, ***p < 0.001.

The mean percentage of the tissue-hyperplasia area, the mean thickness of the submucosal fibrosis, the mean number of epithelial layers, the mean degree of the collagen deposition, and the mean degree of TUNEL- and HSP70-positive deposition were significantly different between the groups (all variables, *p* < 0.001, Kruskal–Wallis test). The mean percentage of tissue-hyperplasia area, mean number of epithelial layers, mean thickness of submucosal fibrosis, and mean degree of collagen deposition were significantly higher in group A than in groups B-D (all variables; *p* < 0.05) (Fig. 3). However, the mean degrees of HSP70- and TUNEL-positive-deposition were significantly lower in group A than in groups B-D (all variables; *p* < 0.05) (Fig. 4). The density grade of inflammatory cell infiltration was not significantly different among the four groups (*p* = 0.726, Kruskal–Wallis test). The mean tissue-hyperplasia area and number of epithelial layers were significantly higher in group C than in groups B and D (all variables; *p* < 0.05). The mean degree of HSP70-positive-deposition was significantly lower in group C than in groups B and D (all variables; *p* < 0.05) (Fig. 5). These histological findings are summarized in Supplementary Table 1.

## Discussion

Our present results have demonstrated that PTT via an AuNP-coated SEMS under NIR laser irradiation will successfully treat granulation tissue formation after stent placement in the rat esophagus. The granulation tissue was significantly decreased in the PTT groups compared to a control group. PTT-induced cell apoptosis was significantly elevated in the heated esophageal mucosa, and markers of cellular proliferation were significantly decreased after PTT when compared to the control animals. Increases in HSP expression and thermal induced-apoptosis are well-characterized features of the heat shock response, and previous studies have reported that HSP70 is an indicator of heat stress in different species^22,23^.

Our study groups were evaluated for the rebound effect. The group C rats treated once at 4 weeks after stent placement had a significant increase in granulation tissue formation at 8 weeks compared to the group D animals treated twice at 4 and 8 weeks after stent placement. The tissue-hyperplasia area and number of epithelial layers in group C were found to have gradually increased compared with the group D rats. Our results confirmed that the PTT was more effective when administered every 4 weeks and indicated therefore that periodic PTT seems to be necessary to treat stent-induced granulation tissue formation after stent placement. Further studies with a long-term follow-up are required to confirm our present findings.

Although PTT has been previously reported to successfully treat granulation tissue formation, there is no consensus regarding the optimal temperature for local heating for the treatment of stent-induced granulation tissue formation^11,20,24–26^. Several studies have reported that at 43 to 65 °C can help inhibit tissue hyperplasia but that an increase to 70 °C induced immediate tissue burn^11,19,25^. In our current study, stent-induced granulation tissue formation in the rat esophagus was effectively treated with AuNP coated SEMS-mediated local heating at 65 °C, which may effectively burn the granulation tissues generated around the stent. Taken together, our present results demonstrated that local heat treatment at 65 °C was optimal for successful PTT.

Our previous studies have reported that an AuNP-coated SEMS can be easily fabricated using simple synthesis steps and is rapidly heated to therapeutic temperatures within a few seconds of NIR laser irradiation^11,20^. The AuNP-coated SEMS used in our current experiments thus rapidly reached a high temperature, which increased in proportion to the NIR power. Hence, the PT properties could be easily controlled by adjusting the NIR irradiation levels. These properties can be attributed to the anisotropic structural characteristics of AuNP, consistent with our prior results^11,20^. Our previous studies involved local heat treatment via the AuNP-coated stent under NIR irradiation at one week after stent placement to prevent stent-induced tissue hyperplasia. In our current study, local heat treatment was performed at 4 weeks after granulation tissue formation had already occurred. Restenosis caused by stent-induced granulation tissue formation occurs as an excessive proliferative response within 4 weeks to the mechanical injury caused by stent placement^10,11,21^. Taken together with previous evidence, our current findings support the notion that local heat treatment via the NIR irradiation of an AuNP-coated stent is an effective therapeutic option for the prevention as well as the treatment of granulation tissue formation after stent placement.

Advances in stent technologies have resulted in a decrease in complications and a prolonged stent patency period. Although a stent has been commonly used as a minimally invasive method to treat malignant and benign esophageal strictures, tissue hyperplasia through the mesh or around the edges of esophageal stents has been reported after the placement of up to 60% of bare stents and 13% of covered stents^27–29^. Hence, permanent stent placement in patients with a relatively long life expectancy has not yet become widespread due to the likelihood of late adverse events, including the development of new strictures caused by stent-induced granulation tissue formation, stent migration, and esophageal ulceration^30,31^. We believe however that our therapeutic strategy using AuNP-coated SEMS could be applied also to an uncovered SEMS to treat granulation tissue growth, and may prolong stent patency by reducing stent-induced tissue hyperplasia, thus improving the patients' quality of life.

There were some limitations to our study of note. First, our findings may not reflect all of the pathological mechanisms occurring in humans following a stent placement. Second, it is necessary to determine the optimal timing for local heating after stent placement to treat stent-induced granulation tissue formation. Third, we did not evaluate the depth penetration of the AuNP-coated SEMS in the rat esophageal model. Although additional studies will be required to further validate our current data, our study supports the premise that PTT via an AuNP-coated stent can successfully treated stent-induced tissue hyperplasia.

An AuNP-coated SEMS appears to be an effective approach for the local treatment of stent-induced granulation tissue formation in the rat esophagus. An AuNP-coated stent-mediated local PTT protocol could be used for not only to treat granulation tissue formation but also tumor ingrowth or overgrowth though the stent meshes. Although further preclinical studies are needed to investigate its efficacy and safety, this therapeutic strategy shows considerable promise for the treatment of granulation tissue formation after stent placement.

## Supplementary information

Supplementary Information.
