# Supplementary material for: Photothermal therapy via a gold nanoparticle-coated stent for treating stent-induced granulation tissue formation in the rat esophagus
Source: Sci Rep. 2021 May 18;11:10558. doi: 10.1038/s41598-021-90182-x (PMC8131374; doi:10.1038/s41598-021-90182-x)
Supplement: Supplementary file 1 — Supplementary Information. [file 41598_2021_90182_MOESM1_ESM.docx]

**SUPPLEMENTAL INFORMATION**

**Photothermal Therapy via a Gold Nanoparticle-Coated Stent for Treating Stent-Induced Granulation Tissue Formation in the Rat Esophagus**

Young Chul Cho, Ph.D.^1†^, Jeon Min Kang, B.S.^2†^, Wooram Park, Ph.D.^3^, Dong-Hyun Kim, Ph.D.^4^, Ji Hoon Shin, M.D.^1^, Do Hoon Kim, M.D.^5*^, Jung-Hoon Park, Ph.D.^2*^

*^1^Departments of Radiology and Research Institute of Radiology, Asan Medical Center, University of Ulsan College of Medicine, 88 Olympic-ro 43-gil, Songpa-gu, Seoul, 05505, Republic of Korea*

*^2^Biomedical Engineering Research Center, Asan Institute for Life Sciences, Asan Medical Center, 88 Olympic-ro 43-gil, Songpa-gu, Seoul, 05505, Republic of Korea*

*^3^Department of Biomedical-Chemical Engineering, The Catholic University of Korea, 43 Jibong-ro, Bucheon-si, Gyeonggi 14662, Republic of Korea*

*^4^Department of Radiology, Feinberg School of Medicine, and Robert H. Lurie Comprehensive Cancer Center, Northwestern University, Chicago, IL, 60611*

*^5^Departments of Gastroenterology, Asan Medical Center, University of Ulsan College of Medicine, 88 Olympic-ro 43-gil, Songpa-gu, Seoul, 05505, Republic of Korea*

^†^Y.C.C. and J.M.K. contributed equally to this work and are the co-first authors.

^*^D.H.K. and J.-H.P. contributed equally to this work and are the co-corresponding authors.

**Correspondence:**

Do Hoon Kim, M.D., Ph.D.^1^ and Jung-Hoon Park, Ph.D.^2^

^1^Department of Gastroenterology, Asan Medical Center, University of Ulsan College of Medicine, 88 Olympic-ro 43-gil, Songpa-gu, Seoul 05505, Republic of Korea

Tel: 82-2-3010-3193 Fax: 82-2-476-0090

E-mail: dohoon.md@gmail.com

^2^Biomedical Engineering Research Center, Asan Institute for Life Sciences, Asan Medical Center, 88 Olympic-ro 43-gil, Songpa-gu, Seoul, 05505, Republic of Korea

Tel: 82-2-3010-4123 Fax: 82-2-476-0090

E-mail: jhparkz[@amc.seoul.kr](mailto:hyjung@amc.seoul.kr)


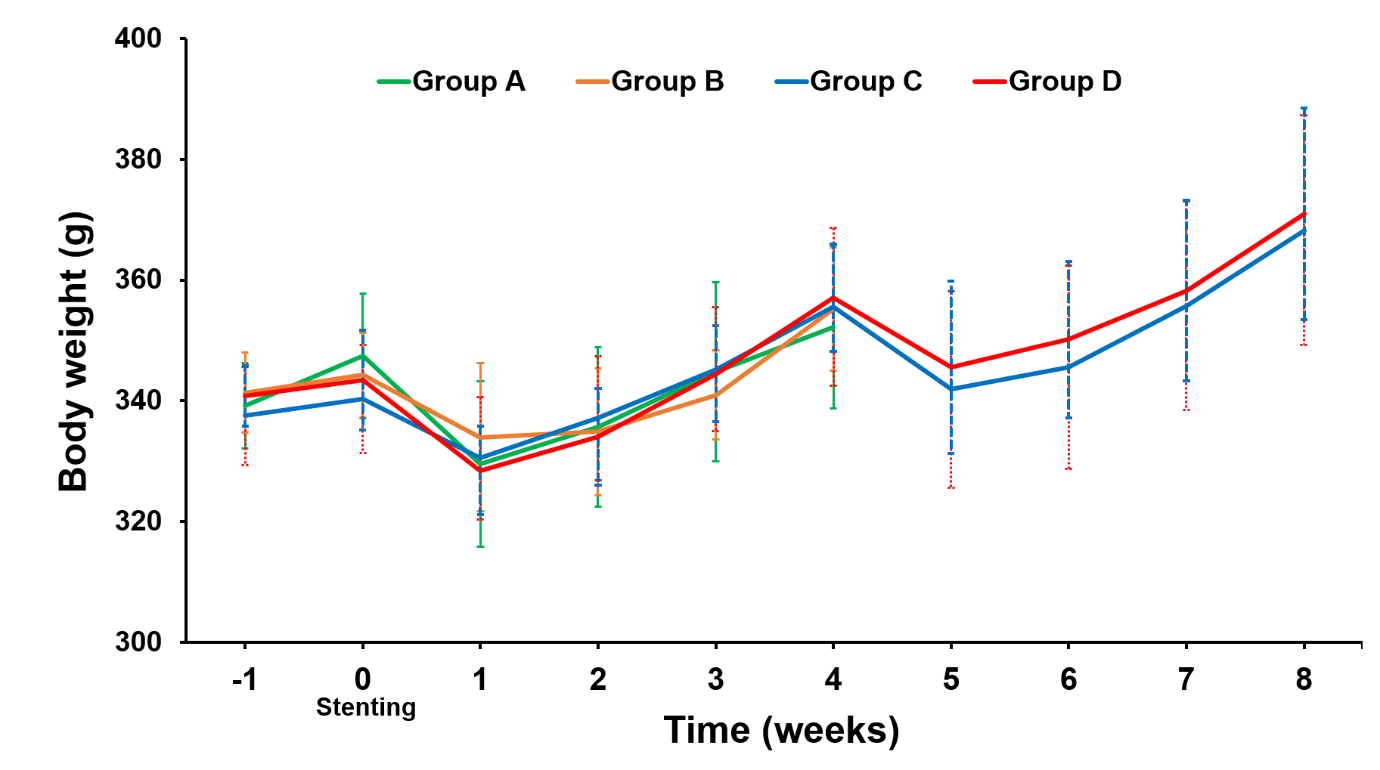


**Supplementary Figure 1.** Effects of stent placement and photothermal therapy on body weight changes.


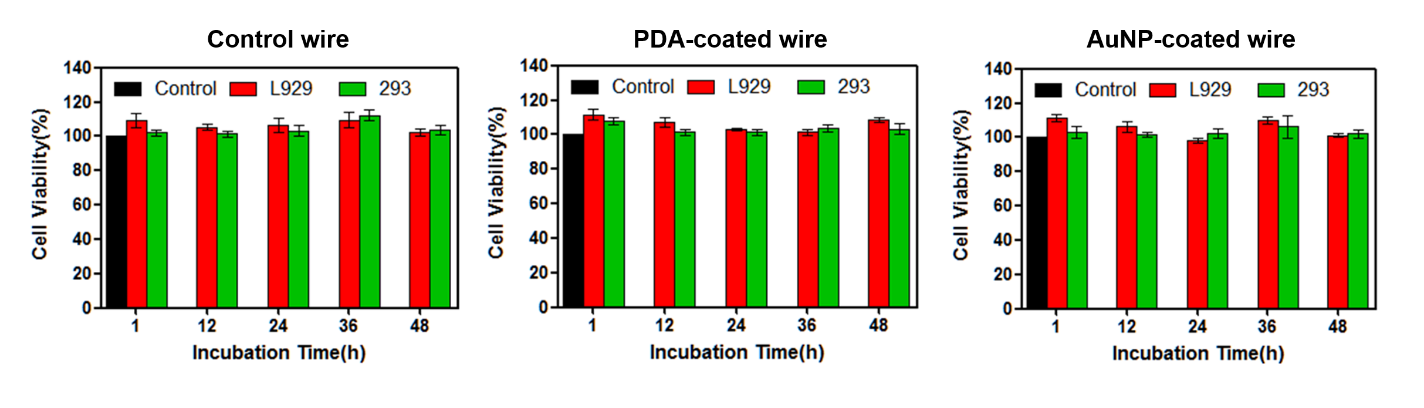


**Supplementary Figure 2.** Cytotoxicity analysis of control, polydopamine (PDA)-coated, and AuNP-coated wire after incubation with L929 and 293 cells at different period of time. Note. PDA, polydopamine; AuNP, gold nanoparticles

**Supplementary Table 1.** Histological findings after local heat treatment when using a gold nanoparticle (AuNP)-coated stent in the rat esophagus

|  | **Group A** | **Group B** | **Group C** | **Group D** | **†*p*-value** | ****p*-value**  **(A vs. B)** | ****p*-value**  **(A vs. C)** | ****p*-value**  **(A vs. D)** | ****p*-value**  **(B vs. C)** | ****p*-value**  **(B vs. D)** | ****p*-value**  **(C vs. D)** |
| --- | --- | --- | --- | --- | --- | --- | --- | --- | --- | --- | --- |
| **HSP70 positive deposition**  **(Grade)** | 1.70±0.73 | 4.05±0.90 | 3.15±0.67 | 4.41±0.67 | < 0.001 | < 0.001 | < 0.001 | < 0.001 | 0.001 | 0.671 | < 0.001 |
| **TUNEL positive deposition**  **(Grade)** | 1.45±0.51 | 3.82±0.80 | 3.85±0.81 | 4.09±0.68 | < 0.001 | < 0.001 | < 0.001 | < 0.001 | 1.000 | 1.000 | 1.000 |
| **Collagen deposition**  **(Grade)** | 3.95±0.60 | 2.50±0.60 | 2.65±0.75 | 2.50±0.67 | < 0.001 | < 0.001 | < 0.001 | < 0.001 | 1.000 | 1.000 | 1.000 |
| **Tissue hyperplasia area**  **(%)** | 52.43±8.88 | 21.80±2.74 | 30.84±5.18 | 22.14±4.44 | < 0.001 | < 0.001 | < 0.001 | < 0.001 | 0.027 | 1.000 | 0.036 |
| **Thickness of submucosal fibrosis**  **(mm)** | 1,232±419 | 348±104 | 489±103 | 375±141 | < 0.001 | < 0.001 | < 0.001 | < 0.001 | 0.432 | 1.000 | 0.870 |
| **Number of epithelial layers**  **(Number)** | 4.35±0.79 | 2.56±0.51 | 3.41±0.51 | 2.61±0.70 | < 0.001 | < 0.001 | < 0.001 | < 0.001 | 0.001 | 1.000 | 0.002 |
| **Inflammatory cell infiltration**  **(Grade)** | 3.16±0.76 | 3.06±0.64 | 3.32±0.75 | 3.17±0.62 | 0.726 | 1.000 | 1.000 | 1.000 | 1.000 | 1.000 | 1.000 |

Note. Data values are a mean ± standard deviations. †Kruskal–Wallis, *Mann–Whitney U test
